# The Role of Fibroblast Growth Factor 10 Signaling in Duodenal Atresia

**DOI:** 10.3389/fphar.2020.00250

**Published:** 2020-03-10

**Authors:** Matthew L. M. Jones, Gulcan Sarila, Pierre Chapuis, John M. Hutson, Sebastian K. King, Warwick J. Teague

**Affiliations:** ^1^F. Douglas Stephens Surgical Research Laboratory, Murdoch Children’s Research Institute, Melbourne, VIC, Australia; ^2^Discipline of Surgery, Sydney Medical School, The University of Sydney, Sydney, NSW, Australia; ^3^Department of Paediatric Surgery, The Royal Children’s Hospital, Melbourne, VIC, Australia; ^4^Department of Paediatrics, The University of Melbourne, Melbourne, VIC, Australia; ^5^Department of Urology, The Royal Children’s Hospital Melbourne, Melbourne, VIC, Australia

**Keywords:** duodenal obstruction, congenital intestinal atresia, fibroblast growth factor 10, fibroblast growth factor receptor 2b, morphogenesis

## Abstract

**Introduction:**

Duodenal atresia (DA) is a congenital bowel obstruction requiring major surgery in the first week of life. Three morphological phenotypes are described, reflecting increasing degrees of obstruction and discontinuity of the duodenum. The cause of DA is not known. Tandler’s original “solid cord” hypothesis conflicts with recent biological evidence, and is unable to account for differing DA types. In humans, a genetic etiology is supported by the association between Trisomy 21 and DA, and reports of familial inheritance patterns. Interruption of FGF10/FGFR2b signaling is the best demonstrated genetic link to DA in mice, with 35–75% of homozygous knockout embryos developing DA.

**Purpose:**

This review examines the current evidence surrounding the etiology of DA. We focus on research regarding FGF10/FGFR2b signaling and its role in duodenal and other intestinal atresia. Further, we outline planned future research in this area, that we consider necessary to validate and better understand this murine model in order to successfully translate this research into clinical practice.

**Conclusion:**

Determining the etiology of DA in humans is a clinical and scientific imperative. *Fgf10/Fgfr2b* murine models represent current science’s best key to unlocking this mystery. However, further research is required to understand the complex role of FGF10/FGFR2b signaling in DA development. Such complexity is expected, given the lethality of their associated defects makes ubiquitous interruption of either *Fgf10* or *Fgfr2b* genes an unlikely cause of DA in humans. Rather, local or tissue-specific mutation in *Fgf10*, *Fgfr2b*, or their downstream targets, is the hypothesized basis of DA etiology.

## Introduction

**FIGURE 1:**
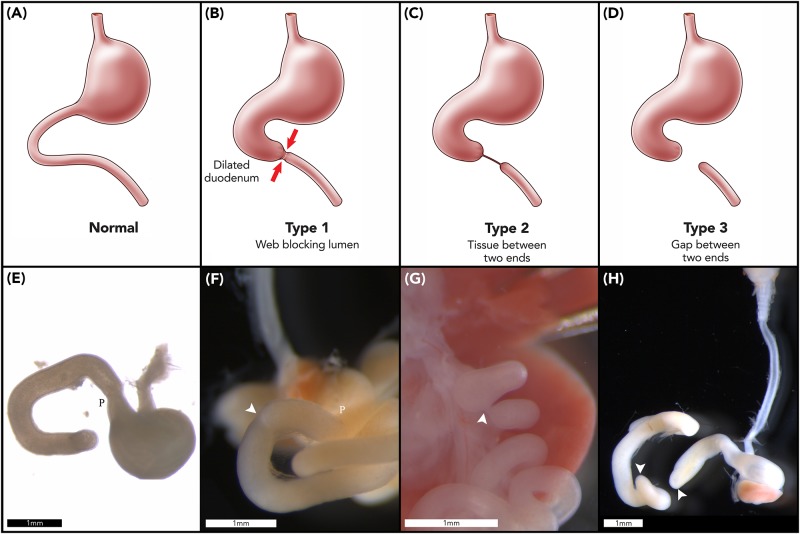
Duodenal atresia phenotype characterization. Normal gastric, pyloric, and duodenal morphology demonstrated pictorially **(A)** and as seen in wildtype murine embryos **(E)**. Type 1 DA is characterized by bowel continuity with luminal obstruction or stenosis **(B)**. Type 2 DA demonstrates bowel discontinuity with a connecting “bridge” of tissue **(C)**. Type 3 DA shows bowel discontinuity with complete separation **(D)**. Our CRISPR-derived *Fgf10* knockout embryos provided examples of type 1 DA **(F)**; type 2 DA **(G)**; and type 3 DA **(H)** including demonstration of an intact esophagus, in the presence of tracheal atresia **(H)**. Annotations denote scale bars, and location of the pylorus, P. Arrows indicate location of an atresia. Figures **(E–H)** reused from Teague et al. (2018), held under the CC-BY 4.0 license.

Duodenal atresia (DA) is a congenital foregut malformation affecting 1 in 5,000–10,000 live births (Escobar et al., 2004). Three morphological types of DA are described, reflecting increasing degrees of obstruction and discontinuity; type 1: bowel continuity but luminal obstruction or stenosis, type 2: bowel discontinuity with a connecting “bridge” of tissue, and, type 3: bowel discontinuity with complete separation (Skandalakis and Gray, 1994; Figures 1A–D). Duodenal atresia was first described in humans by Calder (1733), but it was not until the early twentieth century that Vidal and Ernst performed the first reported successful surgical repair (Vidal, 1905; Ernst, 1916). Nowadays, DA is routinely surgically repaired in the first week of life, with re-establishment of duodenal continuity and minimal postoperative morbidity or mortality (Zani et al., 2017).

The cause of DA is not known. In 1900, Julius Tandler published a hypothesis on the origins of DA, based on his studies of normal duodenal development (Tandler, 1900). Tandler meticulously dissected 11 human embryos and theorized, on the basis of his macroscopic observations, that normal duodenal development includes a “solid cord” phase due to exuberant endodermal growth. As such, he proposed that failure of this “solid cord” to re-canalize may result in DA in humans. Even then, Tandler cautioned readers, stating: “It is clear to me that the opinion represented here does not exceed the status of a new hypothesis, and it is not meant to exceed this.” (Nichol et al., 2011). Notwithstanding, Tandler’s “solid cord” hypothesis is generally accepted worldwide by the majority of pediatric surgeons. This is despite conflicting developmental biology findings (Cheng and Tam, 1998; Botham et al., 2012), and failure of Tandler’s theory to account for the morphological variations of DA (Merrot et al., 2006).

As will be discussed in detail in this review, murine studies have shown that interruption of the *Fgf10-Fgfr2b* signaling axis may result in DA in 35–75% of knockout mice (Fairbanks et al., 2004b; Kanard et al., 2005; Botham et al., 2012; Reeder et al., 2012b; Teague et al., 2018). This also supports a genetic etiology for DA in humans, however, no specific genetic cause has been demonstrated to date. In humans, the association between DA and Trisomy 21 is well recognized, with DA occurring in 20% of cases (Khan et al., 2017). Yet, animal models of Trisomy 21 fail to demonstrate DA or other associated gastrointestinal malformations (Delabar et al., 2006). Again in humans, an autosomal recessive inheritance pattern for familial cases has been proposed, albeit based on a limited number of isolated and historical case reports (Fonkalsrud et al., 1969; Berant and Kahana, 1970; Best et al., 1989; Gross et al., 1996; Lambrecht and Kluth, 1998). Finally, whilst associations between DA and specific chromosomal anomalies have been reported (Mora et al., 2014; Zamfir et al., 2016), none relate to Chromosome 21, or to Chromosome 5 which houses the *FGF10* gene in humans.

## FGF Signaling in Gastrointestinal Development

The mammalian fibroblast growth factors (FGF) family contains 22 genes, 18 of which have been shown to bind and activate tyrosine kinase FGF receptors (FGFR), using either heparin-like molecules or klotho cofactors receptor binding (Ornitz and Itoh, 2015). These 18 FGFs have been grouped into six sub-families based on biochemical function, sequence homology and evolutionary traits (Ornitz and Itoh, 2001). The FGF7 sub-family is comprised of FGF3, FGF7, FGF10, and FGF22, and preferentially binds and activates FGFR2b (the IIIb splice variant of the FGFR2 receptor). This receptor is expressed in the epithelial layer and can be activated by FGF7 and FGF10 ligands (Ibrahimi et al., 2001). These ligands show no binding activity with the mesenchyme-expressed FGFR2c variant (Igarashi et al., 1998; Zhang et al., 2006). Conversely, FGF8 ligand preferentially binds and activates FGFR2c, with no binding activity toward FGFR2b (Macarthur et al., 1995; Ornitz et al., 1996).

**FIGURE 2:**
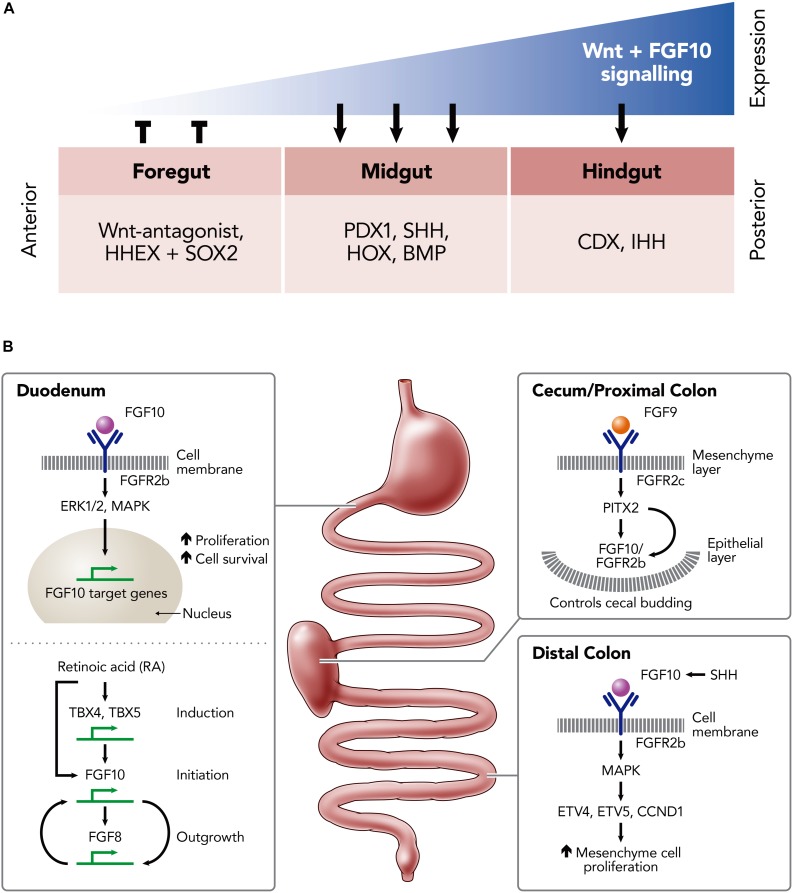
Schematic illustrations of the role of FGF10 in the developing and developed murine intestine. **(A)** The expression-dependent changes of key transcription factors in the foregut, midgut and hindgut mediated by differing levels of FGF10 and Wnt-signaling expression; hematopoietically expressed homeobox protein (HHEX), SRY (sex determining region Y)-box 2 (SOX2) in the foregut; pancreatic and duodenal homeobox 1 (PDX1), Sonic Hedgehog (SHH), homeobox genes (HOX), Bone morphogenetic proteins (BMP) in the midgut; Caudal-Type Homeobox Protein (CDX), Indian hedgehog (IHH) in the hindgut. **(B)** Key FGF10-pathways involved the development of the duodenum, cecum/proximal colon and distal colon; Fibroblast growth factor (FGF) 8, 9, and 10; Tyrosine kinase fibroblast growth factor receptors (FGFR) 2b, 2c; extracellular signal-regulated kinases (ERK1/2); mitogen-activated protein kinase (MAPK); Thromboxanes B4 (TXB4); Thromboxanes B5 (TBX5); ETS translocation variant 4 (ETV4); ETS translocation variant 5 (ETV5); Cyclin D1 (CCND1); Sonic Hedgehog (SHH).

The activation of FGFR2b by FGF10 is associated with mesenchymal/epithelial interactions which instruct branching and budding morphogenesis. This is a key signaling pathway for normal development of the gastrointestinal tract [e.g., stomach (Spencer-Dene et al., 2006), duodenum (Kanard et al., 2005), and large intestine (Burns et al., 2004)] and its derivatives [e.g., lung (Park et al., 1998)]. Indeed, gastrointestinal development involves multiple signaling pathways and their associated transcription factors, including FGF10-FGFR2b, which are summarized in Figure 2A and reviewed elsewhere (Kim and Shivdasani, 2016; Danopoulos et al., 2017).

FGF10-FGFR2b binding is implicated in the activation of multiple intracellular signaling pathways, including the ERK1/2, MAP kinase (MAPK) pathway, to induce downstream activation of proliferation and cell survival genes (Luo et al., 2008; Yang et al., 2011; Figure 2B). TBX4/TBX5 may induce *Fgf10* expression to promote region-specific stimulation of the mesenchymal proliferation at the initial sites of foregut growth. This growth depends on the regulation of the HOX and TBX genes, which are mediated by retinoic acid (RA) (Wang et al., 2006). Potentially, RA acts on TBX genes, such as TBX4 and TBX5, as a co-factor to activate FGF10 transcription and, as a result, mediates the FGF10-FGF8 positive feedback loop which is required for outgrowth (Nishimoto et al., 2015). Embryos that are RA-deficient fail to outgrow due to defective FGF10 signaling, resulting in a lack of FGF-target genes in the epithelium. Interestingly, the development of the foregut may be partly restored in the defective embryos by introducing RA supplementation early on in development (between E7.5 and E8.5), suggesting that RA regulation is upstream of FGF10 (Figure 2B; Wang et al., 2006).

Within the cecum and proximal colon FGF9 binds to the mesenchyme-expressed FGFR2c receptor to direct cecal mesenchyme expansion via the expression of PITX2. In conjunction with this, FGF10 acts to trigger the budding of the epithelium into the cecal mesenchyme, again through mediation of PITX2 expression. Thus, the interaction between FGF10 and PITX2 is critical for the maintenance and the proliferation of the epithelial progenitor cells of the colon (Figure 2B). During embryonic development, FGF10 is also expressed in the mesenchyme of the distal colon, where it binds and activates FGFR1b and FGFR2b to promote proliferation, differentiation and survival of the epithelium. This activation maintains progenitor cells within the embryonic small intestine by targeting E26 transformation-specific (ETS)-Family genes such as ETV4, ETV5, and CCND1 through the MAPK pathway (Nyeng et al., 2011; Figure 2B).

The homozygous deletion of the *Fgf10* or *Fgfr2b* gene results in mice with multiple organ defects, including lung, limb and mammary glands (with failure to form buds 1, 2, 3, and 5). The resultant mice are non-viable after birth due to lung agenesis (De Moerlooze et al., 2000; Mailleux et al., 2002).

## *Fgf10/Fgfr2b* Signaling in Duodenal Atresia

Fairbanks et al. (2004b) were the first to identify a link between the *Fgf10/Fgfr2b* signaling pathway and intestinal atresia in mice. Using *Fgfr2b* knockout mice, they observed DA in 35% of null embryos when examined at gestational stage E18.5. These authors reported examples of both type 1 DA (57%) and type 3 DA (43%), but no type 2 DA (Fairbanks et al., 2004b). The same group then turned their attention to interruption of the ligand instead of receptor, knocking out *Fgf10* (Kanard et al., 2005). Interestingly, *Fgf10* knockout embryos were found to have a similar penetrance of DA (38%) compared to *Fgfr2b* knockouts, but with notably different relative frequency of DA types; type 1 DA (8%), type 3 DA (92%), and still no type 2 DA (Kanard et al., 2005). Further emphasizing the importance of the *Fgf10/Fgfr2b* pathway in intestinal development, deletion of either *Fgf10* or *Fgfr2b* also resulted in a more distally located intestinal atresia, i.e., colonic atresia (Fairbanks et al., 2004a). In contrast with the incomplete penetrance of DA, colonic atresia was present in 100% of *Fgf10* and *Fgfr2b* knockout mice embryos (Fairbanks et al., 2004a).

Importantly, the morphology of the duodenum is normal in *Fgf10* hypomorphic mice, with no examples of DA in this setting of *Fgf10* under-expression (Teague et al., 2018). Thus, even reduced Fgf10 signaling is sufficient for morphologically normal duodenal development. Similarly, Sala et al. (2006) found normal colonic development in *Fgf10* hypomorphic mice, whereas complete loss of *Fgf10* gene function resulted in colonic atresia. In the colon, *Fgf10* expression was shown to be expressed first distally before extending proximally, and was found to be critical for the maintenance and proliferation of the epithelial progenitor cells in the colon. Furthermore, crypt formation seen within the proximal colon in the absence of *Fgf10* suggested other signaling pathways were active in the development of this region, reinforcing the early anatomical and functional regionalization of the GI tract early during development. Another important finding of Sala et al. (2006) was that atresia occured without and associated loss of mesenchyme. This finding makes vascular compromise an unlikely mechanism for colonic atresia in Fgf10 knockouts, as a vascular event would be expected to cause loss of both mesenchyme and epithelium.

The impact of Fgf10 over-expression has also been investigated in both *ex vivo* and *in vivo* models. Torashima et al. (2016) showed that the addition of *Fgf10* to tissue-engineered small intestine (TESI) resulted in increased size and weight of the sample, and increased villi height and crypt depth. In this setting, epithelial differentiation was not inhibited by over-expression of *Fgf10*, a finding attributed to the lack of normal feedback inhibitory mechanisms in the *ex vivo* TESI samples. Nyeng et al. (2011) studied *in vivo* over-expression of *Fgf10* within the developing duodenum. In these embryos, *Fgf10* over-expression caused attenuation of cell differentiation and expansion of the progenitor niche along with upregulation of FGFR signaling targets known to exert feedback inhibition on the FGF pathway (e.g., Sprouty2, Etv4, and Etv5) (Minowada et al., 1999; Nyeng et al., 2011). Conversely, complete loss of *Fgf10* led to the premature differentiation of all enteric cell lineages in the duodenum (Nyeng et al., 2011). These findings support the inference that *Fgf10* is necessary to prevent premature differentiation of enteric cell lineages, and so maintain the epithelial progenitor cell population during development. In extension of this conclusion, these authors hypothesized that premature differentiation and resultant depletion of the progenitor cell population is responsible for the epithelial deficiencies of DA in *Fgf10* knockout mice (Nyeng et al., 2011).

An alternative to the premature differentiation hypothesis, is that proposed by Botham et al. (2012). These investigators concluded that exaggerated endodermal apoptosis, rather than terminal differentiation of progenitor cells was the root cause for DA in *Fgfr2b* knockout mice. Apoptosis was seen in the endoderm at E10.5, disappearance of the endoderm in the atretic region by E11.5, followed by involution of the mesoderm in the absence of further apoptosis at E13.5. This timeline corresponded with first recognition of DA in these *Fgfr2b* knockout mice, also at E13.5. Comparisons were also made between the duodenum of *Fgfr2b* knockout mice and otherwise normal, human embryo samples. In mice, loss of endoderm is complete at an earlier stage of development (E11.5, Carnegie Stage 16). In humans, however, “epithelial crowding” with corresponding apparent loss of duodenal lumen is not seen until Carnegie Stage 17 with “recanalization” only at Carnegie Stage 18. Thus, there is a temporal dislocation between luminal occlusion in atretic murine and normal human duodenal development (Matsumoto et al., 2002; Botham et al., 2012). The timeline for murine DA is supported by the findings of Nichol et al. (2012), who showed murine duodenal narrowing evident from E11.5 and DA from E13.5.

Yet another hypothesis is that of Merei (2004), who proposed that intestinal atresia may be the result of aberrant notochord signaling. Reeder et al. (2012a) used *Fgfr2b* knockout mice, reporting 42% penetrance of DA and 100% penetrance of colonic atresia. These investigators refuted the role of aberrant notochord signaling as the basis for murine DA, showing neither disruption of sonic hedgehog (*Shh*) expression nor notochordal discontinuity in *Fgfr2b* knockouts. Furthermore, organ culture of explanted wildtype murine gut in the presence of supra-physiological concentrations of *Shh* failed to induce atresia in either the duodenum or colon.

One striking feature of the DA reported in the aforementioned, seminal studies is the morphological types of DA reported in *Fgf10* and *Fgfr2b* knockout mice (Fairbanks et al., 2004b; Kanard et al., 2005; Botham et al., 2012; Reeder et al., 2012b). In humans, the distribution of DA types shows similar proportions of type 1 and type 3 DA, and few cases of type 2 DA; e.g., 54% type 1, 4% type 2, and 42% type 3 DA (Khan et al., 2017). In contrast to this, when collated together, 93% of all murine DA cases reported in these studies were examples of type 3 DA, i.e., the most severe type of DA with complete discontinuity of the duodenum (Figure 1D; Fairbanks et al., 2004b; Kanard et al., 2005; Botham et al., 2012; Reeder et al., 2012b). Also, none of these studies reported any examples of type 2 DA in mice.

Of relevance to DA type and the severity of malformation is the work by Reeder et al. (2012b). Reeder et al. (2012b) investigated a role for retinaldehyde dehydrogenase 2 (*Raldh2*), a gene which encodes the enzyme responsible for the final step of vitamin A conversion to RA. *Raldh2* is critical for normal posterior foregut development (Wang et al., 2006), and is under-expressed in the duodenum of *Fgfr2b* knockout mice (Botham et al., 2012). Therefore, Reeder et al. combined heterozygous *Raldh2* deletion with homozygous deletion of *Fgfr2b* with the hypothesis of generating a more severe DA phenotype than with homozygous deletion of *Fgfr2b* alone. Contrary to this hypothesis, addition of *Raldh2* haploinsufficiency resulted in a reduced rather than increased penetrance of atresia (21 vs 45%), and a shift toward less severe atresia types: *Fgfr2IIIb*−/−; *Raldh2*± (80% type 1 DA, 20% type 2 DA, and no type 3) versus *Fgfr2IIIb*-/-; *Raldh2*+/+ (7% type 1 DA, 0% type 2 DA, and 93% type 3 DA). These authors suggested that signaling downstream of *Fgf10/Fgfr2b*, i.e., *Raldh2*, was responsible for the phenotypic variations in DA type morphology.

We have recently reported a more human-like distribution of DA types, including type 2 DA, in two strains of CRISPR-derived *Fgf10* knockout mouse (Teague et al., 2018). Of note, these newly reported strains; tm1 (B6-Fgf10<c.[464_470dup;506_645del]APNMu>); and tm2 (B6-Fgf10<c.495_507delAPNMu>) (Eppig et al., 2006) each demonstrate all three DA types (Figures 1F–H), and a significantly higher penetrance of DA than previous animal models [74% (Teague et al., 2018) versus 35–45% (Fairbanks et al., 2004b; Kanard et al., 2005; Botham et al., 2012; Reeder et al., 2012b). The basis for such differences in type-distribution and penetrance when compared with previously reported *Fgf10* knockout mouse strains remains unclear. The various strains are of consistent background, namely C57/Bl6 (Fairbanks et al., 2004b; Kanard et al., 2005), and whilst the genetics differ, each represents a nonsense mutation (Eppig et al., 2006). We hypothesize that signaling molecules and pathways downstream of *Fgf10-Fgfr2b* are moderating the morphology and penetrance of the DA phenotype in these various strains.

Our hypothesis is supported by the findings of Al Alam et al. (2012). While an FGF9/Pitx2/FGF10 signaling axis has been proposed within the lung (De Langhe et al., 2008), Al Alam et al. demonstrated that the same signaling axis is active in developing gut (Al Alam et al., 2012). Using tissue specific *Fgf9* and *Pitx2* conditional knockout they demonstrated that, within the cecum, FGF9 controls mesenchymal *Pitx2* expression and, in turn, *Fgf10* expression. They also found that *Pitx2* induced the expression of *Fgf10*, even in the absence of *Fgf9*, suggesting that *Pitx2* is downstream of *Fgf9*. In further attempts to define pathways upstream of *Fgf10* involved in atresia, Reeder et al. investigated the role that *Shh* plays with *Fgfr2b* (Reeder et al., 2014). Using a Matrigel culture system and focusing on the colon of *Fgfr2b* knockout mice, they found a downregulation of *Shh* at E11.5 throughout the entire colon, and then loss of *Foxf1* expression 12 h later in the mid-to-distal colon prior to its involution. They also found that the exogenous addition of *Shh* to the *Fgfr2b* deficient colon failed to prevent the formation of atresia, suggesting that the loss of *Shh* is not a critical event in atresia formation.

## Forth-Coming Research Into Duodenal Atresia Etiology

Based on the currently available literature regarding *Fgf10-Fgfr2b* signaling in duodenal atresia, we hypothesize that the etiology of DA in humans is genetically driven, and that the causative genetic changes are downstream of *Fgf10-Fgfr2b*. This downstream locus may account for both the incomplete penetrance of DA in the murine model, and the different morphological types (i.e., severity) of DA. It may also explain why human DA patients lack the non-survivable associations of *Fgf10* deletion such as pulmonary agenesis. Therefore, we plan to test this hypothesis by further investigating the up- and down-regulation of genes and pathways in atretic and morphologically normal duodenum. Coordinated RNA sequencing, validation, and protein interaction analyses are planned, focusing on the duodenum of CRISPR-derived *Fgf10* knockout mice with and without duodenal atresia, as well as additional wildtype controls. We consider that all three of these mice populations are necessary to discern candidate genes and pathways responsible for DA within the murine model. Also, given the complete penetrance of colonic atresia in the setting of *Fgf10-Fgfr2b* signaling interruption, we plan additional experiments to delineate the up- and down-regulation of genes and pathways in atretic and morphologically normal colon. We consider the results of colonic analyses may aid discernment of the genetic changes which hold true relevance for atresia formation, amongst the high volume of genetic changes we expect to observe in this model. Furthermore, we plan to test the aforementioned dose-dependence hypothesis, by scrutinizing gene expression differences between the three morphological types of DA in our CRISPR-derived *Fgf10* knockout mice.

In order to determine the translational relevance of the genetic analysis within our murine DA model, we plan to study the genetic profile of a thought-provoking subset of human DA patients with known associated anomalies akin to Fgf10-Fgfr2b signaling-related defects, e.g., craniofacial, limb, and lung anomalies. We consider this human DA subset to be at particular “risk” of demonstrating an underlying genetic basis for their DA related to the FGF10-FGFR2b signaling pathway. For this analysis, we will screen our institutional DA patient database for case with potential *Fgf10-Fgfr2b* signaling-related defects, and perform detailed clinical genetic phenotyping and exon sequencing of phenotypically homogenous patient groups. During the subsequent bioinformatic and genetic analyses, we will pay particular attention those genes and pathways evidenced to be associated with DA in our murine model.

Ultimate translation of these further studies will be to determine the cause of DA in humans, which will have immediate and therapeutic application in the clinical context of antenatal counseling. Also, these may provide the bases for future hypothesis-driven clinical targets and trials to ameliorate or prevent the development of DA in humans.

## Summary

Interruption of FGF10-FGFR2b signaling is the best demonstrated genetic link to DA in mice. This corresponds in importance, and hypothesis-formulating relevance, to the association between DA and Trisomy 21 in humans. *FGF10* haploinsufficiency and *FGF10* mutations have been linked to human disease, principally to craniofacial syndromes (Entesarian et al., 2005; Milunsky et al., 2006; Rohmann et al., 2006), chronic respiratory disease (Klar et al., 2011), but no human gastrointestinal tract atresia cases to date (Tatekawa et al., 2007). In contrast to this narrative of haploinsufficiency, homozygous *Fgf10* invalidation appears necessary for DA in our animal models (Teague et al., 2018), albeit accompanied by non-survivable anomalies such as lung agenesis. Therefore, we hypothesize that DA is caused by conditional tissue-specific deletion in *Fgf10*, *Fgfr2b* or their downstream targets (e.g., spatially limited to the duodenum or foregut). Constitutional deletion of this nature is less likely to explain DA in humans for reasons of non-survivable anomalies as detailed previously.

We consider determining the etiology of DA in humans to be a clinical and scientific imperative. Therefore, further study is required to understand the exact role of FGF10-FGFR2b signaling in this. Murine models of DA based on interruption of this signaling will be key to unlocking the mystery of DA in humans. Parallel genetic studies of human DA patients will enable translation of model-derived lessons from bench to bedside.

## Author Contributions

MJ conceived the project, conducted the review of literature, analyzed the evidence, and wrote and finalized the manuscript. GS provided specific scientific input into the analysis of evidence, and assisted in writing and finalizing the manuscript. PC mentored MJ and assisted in finalization of the manuscript. JH mentored MJ, and assisted in the analysis of evidence and finalization of the manuscript. SK mentored MJ, and assisted in the review of literature, analysis of evidence, and finalization of the manuscript. WT mentored MJ, conceived the project, and assisted in the review of literature, analysis of evidence, and writing and finalization of the manuscript.

## Conflict of Interest

The authors declare that the research was conducted in the absence of any commercial or financial relationships that could be construed as a potential conflict of interest.
